# Optimizing ChIP-seq peak detectors using visual labels and supervised machine learning

**DOI:** 10.1093/bioinformatics/btw672

**Published:** 2016-11-21

**Authors:** Toby Dylan Hocking, Patricia Goerner-Potvin, Andreanne Morin, Xiaojian Shao, Tomi Pastinen, Guillaume Bourque

**Affiliations:** Department of Human Genetics, McGill University, Montréal, Canada

## Abstract

**Motivation:**

Many peak detection algorithms have been proposed for ChIP-seq data analysis, but it is not obvious which algorithm and what parameters are optimal for any given dataset. In contrast, regions with and without obvious peaks can be easily labeled by visual inspection of aligned read counts in a genome browser. We propose a supervised machine learning approach for ChIP-seq data analysis, using labels that encode qualitative judgments about which genomic regions contain or do not contain peaks. The main idea is to manually label a small subset of the genome, and then learn a model that makes consistent peak predictions on the rest of the genome.

**Results:**

We created 7 new histone mark datasets with 12 826 visually determined labels, and analyzed 3 existing transcription factor datasets. We observed that default peak detection parameters yield high false positive rates, which can be reduced by learning parameters using a relatively small training set of labeled data from the same experiment type. We also observed that labels from different people are highly consistent. Overall, these data indicate that our supervised labeling method is useful for quantitatively training and testing peak detection algorithms.

**Availability and Implementation:**

Labeled histone mark data http://cbio.ensmp.fr/~thocking/chip-seq-chunk-db/, R package to compute the label error of predicted peaks https://github.com/tdhock/PeakError

**Supplementary information:**

Supplementary data are available at *Bioinformatics* online.

## 1 Introduction

Chromatin immunoprecipitation sequencing (ChIP-seq) is a genome-wide assay to profile histone modifications and transcription factor binding sites (Barski *et al.*, 2007), with many experimental and computational steps (Bailey *et al.*, 2013). In this paper we propose a new method for the peak calling step. The goal of peak calling is to filter out background noise and accurately identify the locations of peaks in the genome.

There are two main lines of research into software tools that can help scientists find peaks in the genome. One class of software consists of peak detection algorithms, which are non-interactive command line programs that can be systematically run on all samples in a dataset. An algorithm takes the aligned sequences as input, and returns precise locations of predicted peaks as output. Peak detector software has one major drawback: model selection. There are many different algorithms that have been designed for detecting peaks in specific types of ChIP-seq experiments. Each algorithm typically has several parameters that can be left at default values or changed. Each algorithm and parameter combination will return a different set of predicted peaks. Given a specific ChIP-seq dataset to analyze, how do you choose the best peak detection algorithm and its parameters?

The second class of software consists of graphical tools such as the UCSC genome browser (Kent *et al.*, 2002). To view ChIP-seq data on the UCSC genome browser, the ChIP-seq coverage must be saved to a bigWig file (Kent *et al.*, 2010), which can be browsed as a line or bar plot to visually identify peaks. The main advantage of this approach to peak detection is that it is often easy to visually identify peaks and background noise in coverage plots of ChIP-seq samples. Coverage plots are the standard visual representation of ChIP-seq data (ENCODE Project, 2012; Mikkelsen *et al.*, 2007; Zheng *et al.*, 2010), so it is safe to assume that scientists are capable of visually recognizing relevant peaks in their datasets. However, there are three main disadvantages of this approach. First, precise peak start and end locations are not obvious on visual inspection (at single base resolution). Second, visual interpretation is inherently subjective, and is not exactly reproducible by other researchers. Finally, no researcher has enough time to visually inspect and identify peaks across the whole genome.

**Fig. 1:**
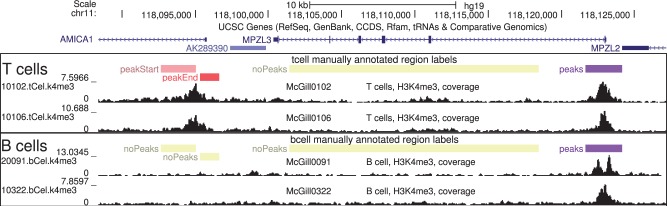
Labels indicate some genomic regions with and without peaks. Visual inspection of ChIP-seq normalized coverage plots was used to create labels that encode where peaks should and should not be detected in these H3K4me3 profiles for T cell and B cell samples. Exactly 1 peak start/end should be detected in each peakStart/peakEnd region. There should be no overlapping peaks in each noPeaks region, and at least 1 overlapping peak in each peaks region

In this article we propose a new supervised machine learning approach for ChIP-seq data analysis that combines the best parts of these two lines of research. The main idea is to manually label peaks in a small subset of the genome, and use those labels to learn a peak detection model that makes consistent predictions on the rest of the genome. In particular, we propose to create labels that encode an experienced scientist’s judgment about which regions contain or do not contain peaks (Fig. 1). The labels can then be used as a gold standard to quantitatively train and test peak detection algorithms on specific datasets. Our proposal contrasts traditional unsupervised peak detection methods, which do not use labels.

### 1.1 Related work: unsupervised peak detectors and benchmarks

The main idea of most existing algorithms for ChIP-seq data analysis is to treat peak detection as a purely statistical problem. Given an assumed model of background noise and peaks, statistical arguments are used to obtain an unsupervised peak detection method. In this paper we limit our study to several such algorithms that predict peaks independently for each sample (Ashoor *et al.*, 2013; Heinz *et al.*, 2010; Kornacker *et al.*, 2012; Song and Smith, 2011; Xu *et al.*, 2010; Zang *et al.*, 2009; Zhang *et al.*, 2008).

A central problem in ChIP-seq data analysis is how to decide which peak detection algorithm to use, and how to choose its parameters. In principle, if the true noise model was known, it would be possible to use statistical arguments to choose the best model and parameters to use. However, in practice the true noise model is complex and unknown. There are many factors that influence the signal and noise patterns in real ChIP-seq data: experimental protocols, sequencing machines, alignment software, etc. Since the noise model in real data is unknown, there is no way to verify the statistical assumptions of an unsupervised model or its default parameters. Thus it is not possible in practice to use statistical arguments to choose an appropriate peak detection algorithm for a given dataset.

Instead of assuming a statistical model, we approach the ChIP-seq peak detection problem using labels and supervised machine learning. Even though the true noise model is unknown in real data, it is still possible to visually label a few genomic regions with obvious signal and noise patterns. These labels provide a well-defined supervised machine learning problem. The best peak calling method can be simply defined as the one which maximizes agreement with the labels, both positive (genomic regions with obvious peaks) and negative (genomic regions containing only noise).

Other existing methods for benchmarking peak detection algorithms include known binding sites (Chen and Zhang, 2010), low-throughput experiments (Micsinai *et al.*, 2012; Osmanbeyoglu *et al.*, 2012) and simulation studies (Szalkowski and Schmid, 2011). Each of these benchmarking methods has its own strengths and weaknesses. For example, known binding sites are useful positive controls for transcription factor ChIP-seq, but are rarely known for histone marks. An unlimited amount of data can be generated using computational simulation studies, but these data may be arbitrarily different from real datasets of interest. Low-throughput experiments are always useful to confirm binding sites at specific genomic locations, but are expensive and slow, so are not routinely done to accompany genome-wide ChIP-seq experiments.

In contrast, the labeling method that we propose requires no additional experiments, so is much more widely applicable. In fact, a similar approach has already been applied to create labeled data for three transcription factors, which have been used to test the accuracy of several unsupervised peak calling methods (Rye *et al.*, 2011). The main novelty of our method is that we also propose to use supervised machine learning with the labels, to train model parameters.

### 1.2 Related work: supervised, interactive analysis

Supervised machine learning methods have been applied to ChIP-seq without using interactive data visualization. For example, a supervised machine learning approach was used to define a regulatory vocabulary with genome-wide predictive power (Gorkin *et al.*, 2012). Another example is several low-throughput experiments which were used to train models for ChIP-seq peak calling (Osmanbeyoglu *et al.*, 2012). In contrast, our proposed method using visually defined labels is much more widely applicable.

Other recent software tools focus on interactive visualization of ChIP-seq data, without using labels and supervised machine learning approaches (Chelaru *et al.*, 2014; Nielsen *et al.*, 2012; Younesy *et al.*, 2013). These are similar to the tools used for Fluorescence-Activated Cell Sorting (FACS) data, where the analyst manually specifies parameters such as fluorescence threshold gates for counting or sorting cells.

Our interactive, visual approach to ChIP-seq data analysis is closely related to several other recently proposed software tools which apply supervised machine learning to biological data with visual labels. For example, CellProfiler Analyst is an interactive system for semi-automatically labeling visually obvious phenotypes in high-content cell microscopy screening assays (Jones *et al.*, 2009). We proposed a similar method for analyzing DNA copy number datasets, using labeled genomic regions with and without obvious breakpoints (Hocking *et al.*, 2013, 2014). The method that we propose in this paper is a first step in the development of an analogous interactive machine learning system for ChIP-seq data.

There are several substantial additions to this paper, with respect to our arXiv pre-print (arXiv:1409.6209). We added results for three previously labeled transcription factor datasets (Rye *et al.*, 2011), two new algorithms (CCAT and PeakSeg) and Receiver Operating Characteristic (ROC) curves for the test error.

## 2 Methods

**Fig. 2:**
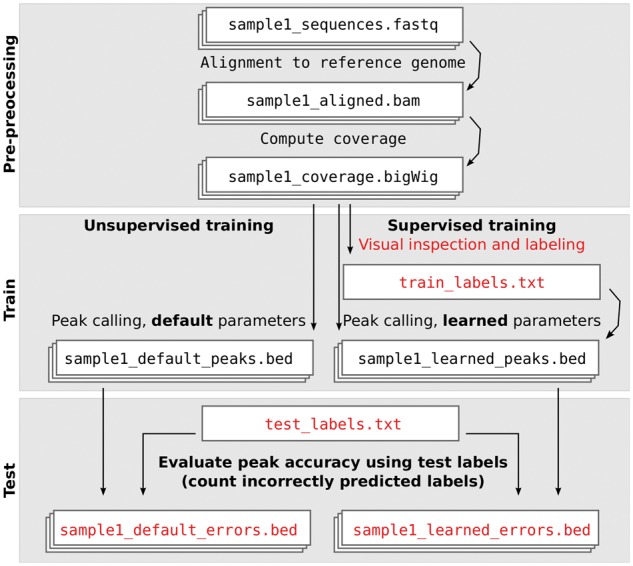
Supervised ChIP-seq analysis includes an extra labeling step. Labels allow learning peak calling parameters during model training, and evaluating peak calling accuracy during model testing

ChIP-seq data analysis pipelines typically contain steps for alignment, coverage computation and peak calling. We propose adding a labeling step between the coverage computation and peak calling steps (Fig. 2). A ChIP-seq pipeline with a labeling step can use supervised machine learning algorithms to compute peak calling model parameters which maximize accuracy with respect to the labels. As we discuss in the Results section, as few as several dozen labels can dramatically improve the accuracy of peak calls.

### 2.1 Labeling samples

Labels can be created by visual inspection of the coverage data in a genome browser black(detailed user guide in Supplementary Text 1). If there are genomic regions that are expected to have relevant peaks (e.g. genes, promoters, predictions from an unsupervised peak caller), begin by labeling those regions. Otherwise, start at a random genomic region, then zoom and scroll through the genome until relevant peaks are visible.

For our proposed labeling method to be meaningful, it is important to label all types of relevant peaks. If peaks with low signal-to-noise ratio are relevant, then make sure to label some. However, to ensure the creation of a high quality set of labels, we recommend to only label genomic regions in which presence/absence of peaks is obvious. We do not recommend labeling any genomic regions in which it is not clear whether or not there is a peak. If the signal-to-noise ratio is so low that there are no visually obvious peaks, then our labeling method should not be used.

For each labeled genomic region, we copied the genomic coordinates to a text file and noted the label type (detailed instructions in Supplementary Text 1). The boundaries of each labeled region can be made as large or small as necessary. We propose to use exactly four types of labels: noPeaks, peaks, peakStart and peakEnd.

- Each **noPeaks** label is used to designate a region that definitely contains only background noise, and contains no peaks.
- A **peaks** label means that there is at least one overlapping peak (of any size). For example, on the right side of Figure 1 it is clear that there is at least one peak, but in profile McGill0091 there seems to be two peaks. So we created a ‘peaks’ label, which means that one or more peaks in that region is acceptable (but zero peaks is unacceptable).
- When a peak start or end is visible, it can be labeled using a **peakStart** or **peakEnd** region. Each labeled region should contain exactly one peak start or end (not zero, and not more than one). If the peak start is clear and the peak end is unclear, then we recommend adding a peakStart region, and not add any nearby peakEnd region. Although it is difficult or impossible to visually locate a precise peak start/end at single base resolution, it is often easy to do so in a larger window. For example Supplementary Figure 2 shows H3K36me3 data in which peakStart/peakEnd regions of size 10–100 kb were used to label peaks which were obviously up, with respect to background noise in adjacent genomic regions. Large peakStart/peakEnd labels can be used to designate a region where there should be just one big peak (and not several smaller peaks, as in Supplementary Fig. 2). For broad domains which may contain more than one separate peak, make sure that the peakStart/peakEnd labels are consistent with the desired peak calls (details in Supplementary Text 1).

When possible, we recommend simultaneous inspection of several samples, to more easily identify common peaks and noise (Fig. 1). For a given experiment type (e.g. H3K4me3), we observed similar patterns in samples of a given cell type, and assigned the same label to each of those samples (e.g. T cells peakStart, B cells noPeaks). The ChIP-seq profiles for samples of interest should be viewed alongside other relevant data such as genes, alignability/mappability, and related input/control samples. If input/control samples are up in a given genomic location, ‘noPeaks’ labels can be used to quantify false positive peak predictions in experimental samples.

So that we could later review and verify the labeled data, we grouped our labels into windows containing several nearby peaks (Fig. 1). We made sure that each window contains at least one noPeaks label and at least one positive label (peaks, peakStart, or peakEnd). Furthermore, we made sure that no label overlaps any other label on the same sample. Usually in machine learning cross-validation experiments, a subset of labeled observations is put aside as a test set. We propose to use windows as the unit of cross-validation, since each window was independently labeled (and labels in each window are not independent). So in our computational experiments, we train on several windows of labels, and test on several other windows.

### 2.2 Label error and peak detection problem

Assume we have *n* labeled training samples, all of the same ChIP-seq experiment type. For simplicity, and without loss of generality, let us consider just one chromosome with *b* base pairs. Let $x_{1}\in {\mathbb{Z}}_{+}^{b},\ldots ,x_{n}\in {\mathbb{Z}}_{+}^{b}$ be the vectors of coverage across that chromosome (counts of aligned sequence reads). For example *b* = 249, 250, 621 is the number of base pairs on chr1, and $x_{i}\in {\mathbb{Z}}_{+}^{b}$ is the H3K4me3 coverage profile on chr1 for each sample i∈{1,…,n}.

**Fig. 3:**
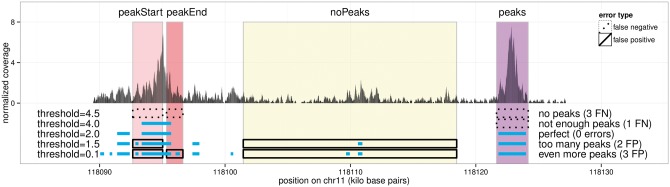
Annotated region labels quantify the accuracy of a peak detection model. Peaks detected by five different thresholds of the HMCan model are shown in blue for tcell sample McGill0102, experiment H3K4me3, labeled by TDH. Models with too few peaks have false negatives (threshold≥4), and models with too many peaks have false positives (threshold≤1.5), so for these data we choose an intermediate threshold = 2 that minimizes the number of incorrect labels

We also have a set of labels *L_i_* for each sample i∈{1,…,n}. Each label $l\in L_{i}$ has a type and an interval of base pairs, e.g. l=(noPeaks,100,200). For example, Figure 3 shows a coverage profile that has one label of each type.

A peak caller is a function $c:{\mathbb{Z}}_{+}^{b}\to {\left\{0,1\right\}}^{b}$ that takes a coverage profile $x\in {\mathbb{Z}}_{+}^{b}$ as input, and returns a binary peak prediction $y=c(x)\in {\left\{0,1\right\}}^{b}$ (0 is background noise, 1 is a peak).

The goal is to learn a peak calling function c(x) which is consistent with the labeled regions *L*. To quantify the error of the peak calls with respect to the labels, we define the error *E* as the total number of false positive (FP) and false negative (FN) labels:

(1) E(y,L)=FP(y,L)+FN(y,L)∈{0,1,2,…,|L|}.

The principle used to compute the label error *E* is illustrated in Figure 3, and the precise mathematical definitions of FP and FN are given in Supplementary Text 3. In short, a false positive occurs when too many peaks are predicted in a labeled region, and a false negative occurs when there are not enough predicted peaks. The number of incorrect labels E(y,L) is at least 0 (when all labels are correctly predicted) and at most |L| (when all labels are incorrect). In practice we recommend using the PeakError R package (https://github.com/tdhock/PeakError), which contains functions for computing the label error *E*.

The supervised machine learning problem can be formalized as follows. Find the peak caller *c* with the minimum number of incorrect labels when calling peaks in an un-seen test dataset:

(2) $$\underset{c}{\min\mathrm{imize}}\sum_{i\in \mathrm{test}}E\left[c\left(x_{i}\right),L_{i}\right].$$

In the Results section, we consider several types of test sets to answer different questions using computational cross-validation experiments (Table 1). For example, in one case we train on labels from one person and test on labels from another person, to show that different people have consistent definitions of peaks.

**Table 1:** Examples of different training and test sets that we used to answer different questions using computational cross-validation experiments

Figure	Same	Train	Test
Figure 5	person, cell types, experiment	chr1	chr2
Supplementary Figure 5	cell types, experiment	labels from TDH	labels from PGP
Supplementary Figure 7	person, experiment	T cells	kidney cells
Supplementary Figure 8	person, cell types	H3K4me3	H3K36me3

For example, Supplementary Figure 7 shows that a model trained on immune cells provides accurate peak calls for samples of different cell types.

## 2.3 Training unsupervised peak detection models

Most existing peak detection models are ‘unsupervised’ in the sense that they are not designed to be trained using the labeled regions that we propose. However, in this section we show that grid search over one of their parameters can be used as a model training procedure.

We considered several unsupervised peak detection models with free/open-source software implementations (details in Supplementary Text 2). MACS, HMCan, and CCAT have parameters for sharp peaks and broad domains, so we tried both settings (Ashoor *et al.*, 2013; Zhang *et al.*, 2008). RSEG and SICER were designed for broad domains (Song and Smith, 2011; Zang *et al.*, 2009). The HOMER set of tools contains a findPeaks program which has been used to detect transription factor binding sites and histone modifications (Heinz *et al.*, 2010). The Triform algorithm was shown to have good peak detection for transcription factor ChIP-seq experiments (Kornacker *et al.*, 2012).

Each model has several parameters that may affect peak detection accuracy. Some parameters may have a large effect on peak calls, whereas other may have smaller effects. An exhaustive grid search over several parameters would be infeasible, since there is an exponential number of different parameter combinations (Micsinai *et al.*, 2012). So for each model we train by calibrating a single significance threshold parameter, and holding other parameters at default values. In the Results section we show that this single parameter grid search is sufficient to improve peak detection accuracy of most algorithms. Further improvements could be made by applying heuristic search algorithms over the multi-dimensional parameter space, which we leave for future work.

**Fig. 4:**
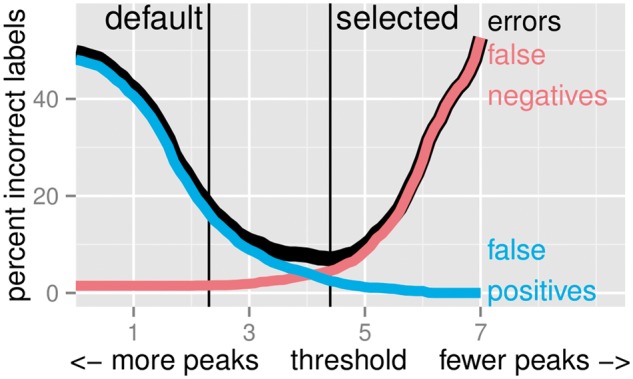
Peak detectors can be trained by selecting model parameters that minimize the number of incorrect labels. We plot the percent error as a function of the finalThreshold parameter of the HMCan.Broad model in the 1743 labels of the H3K36me3_AM_immune dataset

Each algorithm can thus be described as a function $c_{\lambda }:{\mathbb{Z}}_{+}^{b}\to {\left\{0,1\right\}}^{b}$, where the significance threshold parameter λ∈ℝ controls the number of peaks detected. In each peak detection algorithm, *λ* has a different, precise meaning that we specify in Supplementary Text 2. Typically, low thresholds *λ* yield too many peaks, and high thresholds *λ* yield too few peaks (Fig. 3). As shown in Figure 4, we select an optimal threshold *λ* by minimizing the total label error *E* in the set of *n* training samples

(3) $$\hat{\lambda }=\underset{\lambda }{\text{arg min}}\sum_{i\in \left\{1,\ldots ,n\right\}}E\left[c_{\lambda }(x_{i}),L_{i}\right].$$

The training or model calibration procedure (3) consists of simply computing peak calls for several peak detection parameters *λ*, and choosing whichever parameter $\hat{\lambda }$ minimizes the number of incorrect labels (formally, this algorithm is called grid search). To simulate the case of an unsupervised ChIP-seq pipeline (no labels available), we can simply use the default significance threshold $\tilde{\lambda }$ suggested by the author of each algorithm. The test error (2) can be used to evaluate the accuracy of the trained model $\hat{\lambda }$ and the default model $\tilde{\lambda }$.

### 2.4 The supervised PeakSeg model

We also considered the supervised PeakSeg model, which was shown to have state-of-the-art peak detection accuracy in both sharp H3K4me3 data and broad H3K36me3 data (Hocking *et al.*, 2015). Unlike standard unsupervised models which can only be trained using grid search, PeakSeg was designed to be trained using labels. The PeakSegDP R package (https://github.com/tdhock/PeakSegDP) implements discrete and convex optimization algorithms to find model parameters which maximize peak detection accuracy with respect to a set of labels.

## 3 Results

### 3.1 Creating labeled histone datasets

Since the only other labeled benchmark dataset is limited to transcription factors (Rye *et al.*, 2011), we decided to label samples from two histone mark experiments. We analyzed ChIP-seq samples from the McGill Epigenomes portal (http://epigenomesportal.ca), which we visualized using the UCSC genome browser software (Kent *et al.*, 2002). We labeled 37 H3K4me3 samples (sharp peak pattern) and 29 H3K36me3 samples (broad peak pattern). In these data there are many samples of the same cell type, with 8 distinct cell types (Supplementary Table 1). The same peaks often occur in all samples of the same cell type (Fig. 1).

We constructed the 7 labeled histone mark datasets using the method described in the ‘Labeling samples’ section. Of the four different people who created the labels, some were post-docs (TDH, XJ), and some were PhD students (AM, PGP). In total we created 12 826 labels, in genomic windows with on average 1–4 peaks per sample (Supplementary Table 2). The datasets are published to a public web site (http://cbio.ensmp.fr/∼thocking/chip-seq-chunk-db/), and we will refer to them as the McGill ChIP-seq peak detection benchmark datasets.

We are only aware of one other publicly available benchmark with labels, a set of three transcription factor ChIP-seq datasets from NTNU (Rye *et al.*, 2011). There are several differences between these datasets and our McGill benchmark datasets. First, each experiment in the NTNU benchmark includes two replicate samples with the same expected peak pattern. In contrast, each McGill dataset includes several samples of different cell types, so some labels indicate only certain cell types have a peak in a given genomic region. For example Figure 1 shows a genomic region with a labeled peak in T cells but not B cells. Another difference is that the NTNU datasets were labeled by visual verification of regions with called peaks. In contrast the McGill datasets were labeled by visual inspection of random genomic regions, before running any peak calling algorithms.

### 3.2 Trained models are more consistent with test labels than default models

We expected that default model parameters are not always consistent with the labels. In agreement with our expectation, we observed that training a significance threshold parameter can reduce the number of incorrect labels, with respect to default parameters. For example, Supplementary Figure 1 shows an H3K4me3 sample for which the default parameter of MACS yields a false positive, but the trained parameter yields perfect agreement with the labels. As another example, Supplementary Figure 2 shows an H3K36me3 sample for which the default parameter of HMCan.Broad yields two false positive labels, but the trained parameter yields perfect agreement with the labels.

**Fig. 5:**
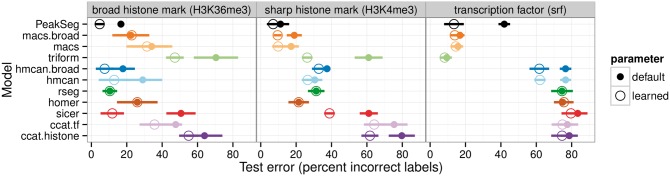
Model parameters which are learned using labels provide more accurate peak predictions than default model parameters. Four-fold cross-validation was used to estimate test error rates (mean ± standard deviation over four test folds). Closed circles represent default parameters (labels not used for model training), and open circles represent learned parameters (with minimal incorrect labels in each training dataset). It is clear that some algorithms are accurate in several data types and others only work in one data type

To quantitatively compare peak predictions using trained and default model parameters, we performed 4-fold cross-validation in each of the 10 labeled datasets. We randomly assigned each labeled genomic window to one of four folds. For each fold, we treat that data subset as a test set, and train models using all other folds. Then we compute the number of incorrect regions in the test fold. In general it is clear that test error rates are lower with learned parameters than with default parameters (Fig. 5). Over all 11 algorithms and all 40 test folds, the average improvement was 9.4±2.3% incorrect labels (mean ± 95% *t*_439_ confidence interval). The only exception is that for some models such as rseg, the test error of the learned parameter is the same as the test error for the default parameter.

These data suggest that labels can definitely be used to train model parameters for increased peak detection accuracy.

### 3.3 Different algorithms are appropriate for different experiment types

Since different ChIP-seq experiments have different peak patterns (e.g. sharp versus broad histone marks), we expected that each unsupervised algorithm would provide accurate peak detection for only one experiment type. We expected the supervised PeakSeg algorithm to adapt to the labels in each dataset, and provide accurate predictions for each experiment type.

In agreement with our expectation, we observed that unsupervised algorithms yield accurate peak predictions in only certain experiments (Fig. 5). For example, Triform yields accurate predictions in transcription factor datasets but not histone marks. Also, MACS yields better predictions for transcription factor and sharp histone mark data than for broad histone mark data.

We observed that the supervised PeakSeg method yields state-of-the-art accuracy in most of the datasets we analyzed (Supplementary Fig. 3). The only exception is that Triform was more accurate for the transcription factor datasets. These results confirmed our expectation that since PeakSeg was designed to be trained using labels, it can adapt to different labeled patterns, and provide accurate predictions for almost any type of experiment.

We computed Receiever Operating Characteristic (ROC) like curves for the test error of each algorithm. These ROC-like curves plot true positive and false positive rates for a range of peak calling parameters. Consistent with the qualitative examples in Supplementary Figures 1 and 2, the ROC-like curves quantitatively show that default parameters tend to yield higher false positive rates than learned parameters (Supplementary Fig. 4). It is also clear that some algorithms are more accurate than others, for all possible thresholds *λ*. In particular it is clear that PeakSeg is more accurate than most other methods for all possible thresholds *λ*.

### 3.4 Only a few labels are required to train an accurate model

One limitation of our proposed method is that to perform labeling, some time is required for manual visual inspection of the ChIP-seq data. However, we show in this section that our method is useful even if there is only time to label a few genomic regions.

**Fig. 6:**
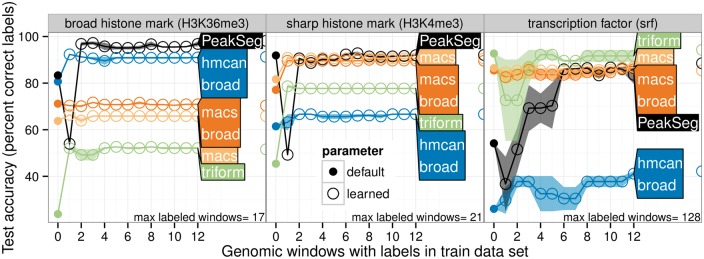
Only a few labels are required to train an accurate model. Some labeled genomic windows were set aside as a test set, then models were learned for each training set size, for two different random orderings of the training set (mean line and min/max band). A circle on the right shows the test error of the model trained with the maximum number of windows. It is clear that in each dataset, the model-specific maximum accuracy is achieved after only 2–6 labeled windows (several dozen labels)

To determine how many labels are necessary to learn a model with maximum accuracy, we fixed a test fold, and then considered training models using a variable number of labeled genomic windows from the training folds (from 0 to 12). We observed that each algorithm quickly achieves its model-specific maximum accuracy (Fig. 6), after only about 2–6 labeled windows in the training set (several dozen labels).

### 3.5 Labels from different people are highly consistent

One of the fundamental assumptions of our method is that a researcher is capable of consistently labeling visually obvious peaks and noise in ChIP-seq coverage plots. However, labels from a particular researcher are inherently subjective, and we expect that labels from different people will not always agree. However, since peaks and noise are often very obvious when visually inspecting coverage plots of several ChIP-seq samples, we also expect labels from different people to be highly consistent.

To determine the extent of consistency between people, we considered training each peak detector using labels from one person, and testing them on labels from the same or a different person (holding experiment and samples constant, second row of Table 1). We observed very little changes in test error when training on one or the other person (Supplementary Fig. 5). Over all algorithms and all test folds, the average difference in test error was not significantly different from zero (0.08±0.16% incorrect labels, mean ± 95% *t*_87_ confidence interval). The algorithm with the largest difference was PeakSeg, which sometimes had higher test error when testing on another person (2.0±4.1%, mean ± 95% *t*_7_ confidence interval). More generally, it is important to realize that although the learned peak calls were highly consistent between different people, they are not exactly reproducible (e.g. it is unlikely that the optimal parameters for two people are exactly the same).

Also, there were some genomic windows in the H3K36me3 immune datasets that were labeled by both TDH and AM. It is clear that these two sets of labels are consistent, but provide different levels of detail. For example, TDH used a peakStart and peakEnd label in many instances where AM used a peaks label (Supplementary Fig. 6). Also, there are other regions which only TDH labeled, and AM left un-labeled.

Overall, these data indicate that the outcome of comparing models is consistent using labels from different people. Labeled regions can thus be used as a method for benchmarking peak detection algorithms, with results that are specific to the ChIP-seq dataset, and quite robust across labels from different people.

### 3.6 Trained models predict accurate peaks in samples of the same experiment

In real ChIP-seq datasets, there are often dozens or even hundreds of samples to analyze. If there is not enough time to label all the samples, we expect that training a model on a subset of labeled samples should yield accurate predictions on the other un-labeled samples (for the same experiment type, e.g. H3K4me3). To evaluate the extent to which trained models generalize to other samples, we trained each algorithm on several immune cell samples and tested them on samples of other cell types (holding person and experiment constant, third row of Table 1).

We observed that most algorithms had similar test error when training on the same or different cell types (Supplementary Fig. 7). The exceptions were the MACS and MACS.Broad algorithms, which exhibited higher test error when training on samples of different cell types. These data suggest that the MACS qvalue parameter does not generalize well between different samples. For accurate peak detection using the MACS algorithm, these data suggest to label all samples of interest, and then learn sample-specific qvalue parameters.

### 3.7 Trained models do not predict accurate peaks in other experiments

Since different experiments can have very different peak patterns, we did not expect that models trained on one experiment would work on other experiments (e.g. sharp versus broad histone marks). To quantitatively demonstrate this point, we trained each algorithm on one histone mark and tested them on another (holding person and samples constant, last row of Table 1). In agreement with our expectations, we observed that all algorithms had higher test error when trained on a different experiment (Supplementary Fig. 8).

## 4 Discussion

### 4.1 Supervised versus unsupervised analysis

In the machine learning literature, a problem is considered supervised when there is a teacher or expert that provides correct predictions for a learning algorithm. In this paper, the type of supervision that we proposed was a database of labeled regions that represents where a scientist does and does not observe peaks. We used these labeled regions as a gold standard to define a prediction problem (2), which seeks a peak detector with minimal incorrect labels on a test dataset. Furthermore, we proposed choosing a peak detection parameter $\hat{\lambda }$ (3) that minimizes the number of incorrect labels in a training dataset.

In contrast, ChIP-seq peak detection without labeled regions can be considered an unsupervised learning problem. Usually, an algorithm with default parameters $\tilde{\lambda }$ is first fit to a dataset, and then peaks are qualitatively judged by visualizing them along with the data in a genome browser. The user can manually change the parameters *λ*, until the predicted peaks appear to be satisfactory (this is human learning, not machine learning).

In fact, our supervised analysis protocol with labeled regions is similar, but is independent of any specific peak detection algorithm. First, one must visually inspect the ChIP-seq data in a genome browser, and label some genomic regions with and without peaks. After that, those labels can be used to train model parameters $\hat{\lambda }$, and test different models in particular ChIP-seq datasets of interest.

Our supervised analysis protocol is a user-friendly alternative to manual parameter tuning of an unsupervised peak caller. In fact, manual parameter tuning only works when the user knows how the parameters *λ* affect the output peaks. However, users do not often understand the details of peak calling parameters, so typically leave the parameters at default values. In contrast, our supervised method exploits the fact that it is often easy to label peaks when visualizing ChIP-seq data in a genome browser. After labeling a few genomic regions with and without peaks, the labels can be used for automatically selecting optimal peak calling parameters $\hat{\lambda }$.

### 4.2 Time required for labeling

One potential criticism of our proposed method is that it requires a new labeling step (Fig. 2), which may be time-consuming. However, in our experience, this labeling step requires a relatively small time investment. It only takes a few minutes to find and label a whole window of several nearby genomic regions. This is an insignificant amount of time compared to the time required to perform experiments, write code for data analysis, and write papers.

Additionally, we were able to quickly create many labels by simultaneously visualizing dozens of samples. For example, the immune H3K4me3 sample set consists of 27 samples. When we found a region with a peak across all samples, we assigned the same label to all 27 samples.

As another example, it only took about 40 min to create the entire H3K36me3_TDH_other dataset, which contains in total 8 samples and 200 labeled regions across 4 genomic windows. And even though this was the smallest dataset that we created, we were still able to observe clear differences in test error between the various algorithms (Supplementary Fig. 3).

Also, the test accuracy curves indicate that only a few labeled genomic windows are necessary to train algorithms (Fig. 6). In all datasets, the test accuracy increases to its model-specific maximum after labeling only about 2–6 genomic windows (several dozen labels).

Overall, these data indicate that a relatively small number of labels allows both training model parameters and testing different peak detection algorithms. Thus, it is quite reasonable to spend a few minutes creating labels as a part of a supervised ChIP-seq data analysis pipeline.

### 4.3 Advice for choosing a peak caller and parameters

Practically speaking, which peak caller and what parameters should be used for a given dataset? We have proposed a general method for quantitatively answering that question. First, label a few genomic regions, and divide the labels into training and test sets. Then, run several peak callers, using the labels in the training set to choose model parameters. Finally, compare predicted peaks with the labels in the test set. The most accurate peak caller has the minimum number of incorrectly predicted test labels.

If you do not have time to create any labels, our data suggest using a different algorithm for each experiment. For example, we observed that Triform is accurate in transcription factor data but not histone data, and that MACS is accurate in sharp H3K4me3 histone data, but not broad H3K36me3 data (Fig. 5). However, beware that default parameters typically yield high false positive rates (Supplementary Figs. 1 and 4).

## 5 Conclusions

We proposed a supervised machine learning approach for ChIP-seq data analysis. Our approach involves first using visual inspection to label regions with and without peaks in a specific dataset, and then using those labels to train and test peak detection models.

We used this approach to benchmark the performance of several peak detectors on several labeled histone mark and transcription factor datasets. We observed that while some unsupervised methods yield accurate peak calls for specific experiments, the supervised PeakSeg method was the most accurate in general across all experiments.

We limited our study to single-sample peak calling algorithms. In the future, we will be interested to use visually defined labels for training and testing multi-sample peak detection algorithms such as JAMM and PePr (Ibrahim *et al.*, 2014; Zhang *et al.*, 2014).

We have made available several labeled histone samples as a public benchmark dataset. Such labeled datasets are essential for making links between computational biology and the larger computer science research community. In particular, these labeled datasets make the ChIP-seq peak detection problem more accessible to machine learning researchers, who will now be able to work on developing more accurate supervised learning algorithms for peak detection.

Our results indicated that models learned on one of our benchmark datasets yield accurate predictions for our other datasets of the same experiment type. In the future, we would also like to examine the extent to which these models generalize to ChIP-seq data from other labs, using different experimental protocols, and different alignment programs.

Finally, we are interested in developing an interactive labeling and machine learning web application for ChIP-seq data, possibly using Apollo (Lee *et al.*, 2013). An ideal system for interactive ChIP-seq data analysis would display current peak predictions on the genome browser, and allow the user to correct any obvious errors by labeling genomic regions with and without peaks. Then the system would learn from those labels, and automatically update the peak calling model in order to provide more consistent predictions.

## Funding

This work was supported by computing resources provided by Calcul Quebec and Compute Canada, Natural Sciences and Engineering Council of Canada RGPGR 448167-2013, and by Canadian Institutes of Health Research grants EP1-120608 and EP1-120609, awarded to GB.

*Conflict of Interest*: none declared.

## Supplementary Material

Supplementary Data
