# Supplementary material for: Optimizing ChIP-seq peak detectors using visual labels and supervised machine learning
Source: Bioinformatics. 2016 Nov 21;33(4):491–9. doi: 10.1093/bioinformatics/btw672 (PMC5408812; doi:10.1093/bioinformatics/btw672)
Supplement: Supplementary Data [file btw672_supp.pdf]

Supplementary materials for "Optimizing ChIP-seq peak detectors using visual labels and supervised machine learning"

Toby Dylan Hocking (toby.hocking@mail.mcgill.ca),
Patricia Goerner-Potvin, Andreanne Morin, Xiaojian Shao, Tomi Pastinen,
and Guillaume Bourque (guil.bourque@mcgill.ca)

October 7, 2016

Supplementary Text 1

User guide on how to create labels. This section gives a detailed explanation of how to create labels for ChIP-seq data sets.

Tracks to visually inspect. Start by viewing all relevant tracks at the same time in a genome browser:

- Counts of aligned reads for several samples of a single type of ChIP-seq experiment. For example, if you have both H3K4me3 and H3K36me3 data, then start by visualizing and labeling just the H3K4me3 samples. If you are interested in differences between samples or cell types, make sure to include all the different sample types (e.g. both knock-out and wild-type samples).
- Counts of aligned reads for negative control ChIP-seq samples (also known as background or input samples). These samples are important in order to determine which peaks are specific to the antibody used in the ChIP-seq experiment. Non-specific peaks appear up in both experimental and negative control samples.
- Other genome browser tracks such as alignability/mappability and GC content. These tracks may influence how to interpret the aligned read coverage signal in terms of peaks and background.
- Peak calls can be displayed in order to visualize peak calling errors that need correction via labels.
- Labels can be displayed in order to see which genomic regions have already been labeled.

If your computer screen is too small to display all relevant tracks, then labeling can be used on subsets of the data. For example, if there are 500 ChIP-seq samples to analyze, we would recommend starting by visualizing and labeling a subset of 10–30 samples. If you are interested in differences between samples, make sure that the subset contains examples of several different sample types, so you can observe and label differences between sample types. For example, the figure below shows 27 samples of three cell types (bcell, tcell, monocyte).

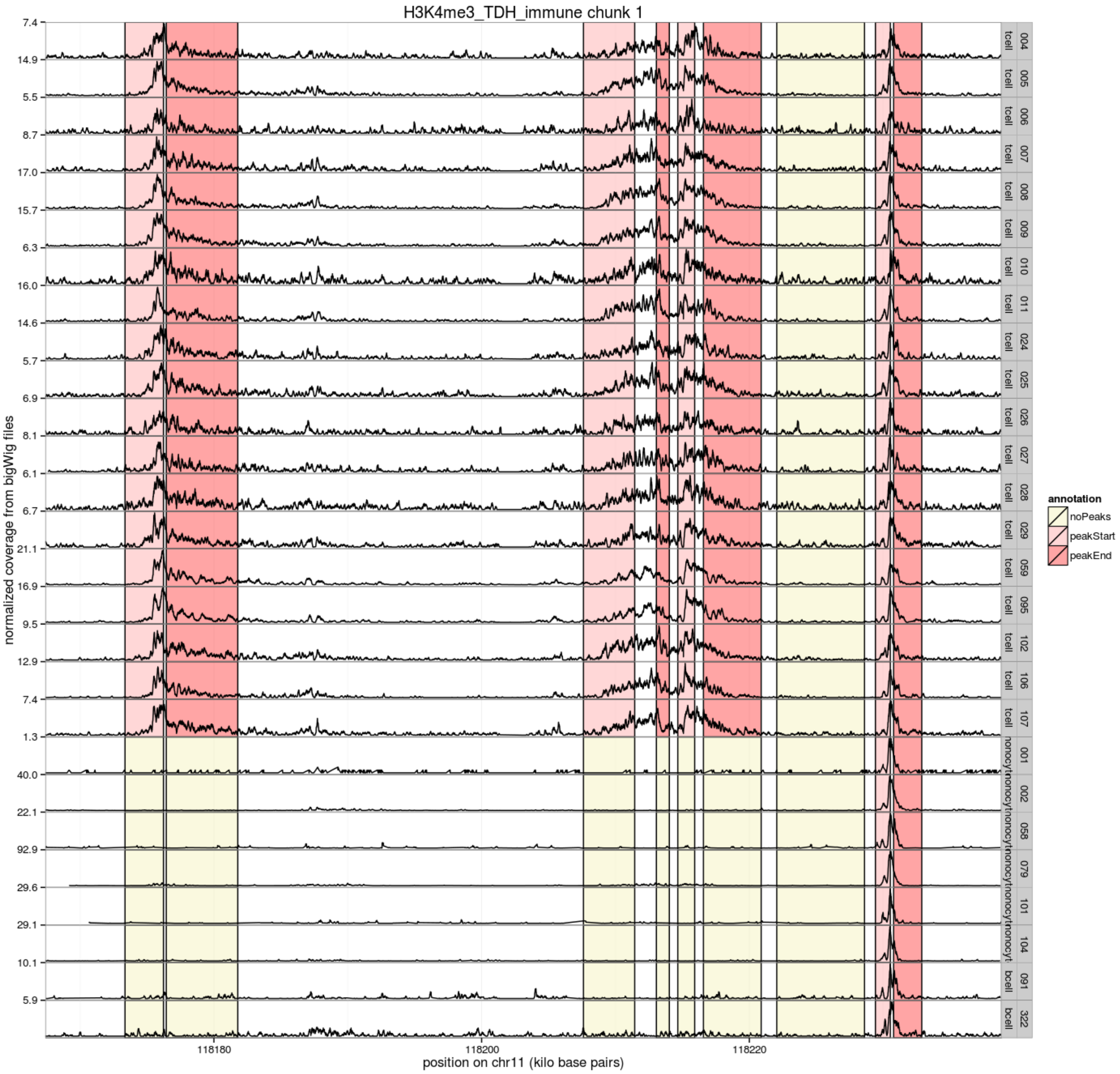

Genome browser axis settings. Typically genome browsers have two options for the display of the y-axis scale. If all samples are of the same sequencing depth (for example 10x) and are expected to have about the same amount of coverage, then the y axis can be forced to have the same scale across all samples. For samples sequenced at different depths (for example 5x and 50x), the y-axis should be set to automatically rescale to the maximum of each sample. For example, this autoscale setting is useful to see peaks in background in the figure above (the normalized coverage counts range from 5 to 93, but peaks and background are still visually obvious when the y axis is autoscaled).

Choosing genomic regions to label. There are several methods that can be used to find genomic regions to label:

- If you expect peaks in certain genomic regions (e.g. genes, promoters), then you can start by looking in those parts of the genome.
- If you have preliminary peak calls for the samples you want to label, then you can view a genome subset with peaks that have certain properties (e.g. small p-value, large length, large height).

- Otherwise, you can start in a random region of the genome, and then zoom in or out until you locate some peaks that are visually obvious.

Continue browsing through the genome until you are confident to have labeled an unbiased, representative subset of relevant peaks.

Grouping nearby labels into windows. We recommend creating “labeled windows,” which are genomic regions with groups of nearby labels. The reason we create windows is in order to make it easy to visually check the labels, after labeling has been performed. It is not easy to visually check a single label by itself in the genome. For example a positive peaks label may not be obvious unless the genome browser is zoomed out enough to see the nearby background noise (and perhaps a negative noPeaks label). Therefore, each labeled window should

- Contain at least one region where all samples have a positive label (peaks, peakStart, peakEnd). Thus the maximum of an autoscaled y axis will equal the maximum coverage of a peak.
- Contain at least one region where all samples have a negative label (noPeaks). Thus the contrast between peaks and background will be easy to verify.
- Ideally contain at least one genomic region which has positive labels for some samples, and negative labels for the other samples. These labels further clarify the definition of a significant difference between peaks and background.

Examples of labeled windows that satisfy all three criteria are shown in the figures above and below. Since the labels in each figure constitute a single window, it is easy to verify them by simply plotting the coverage data and labels in the entire window.

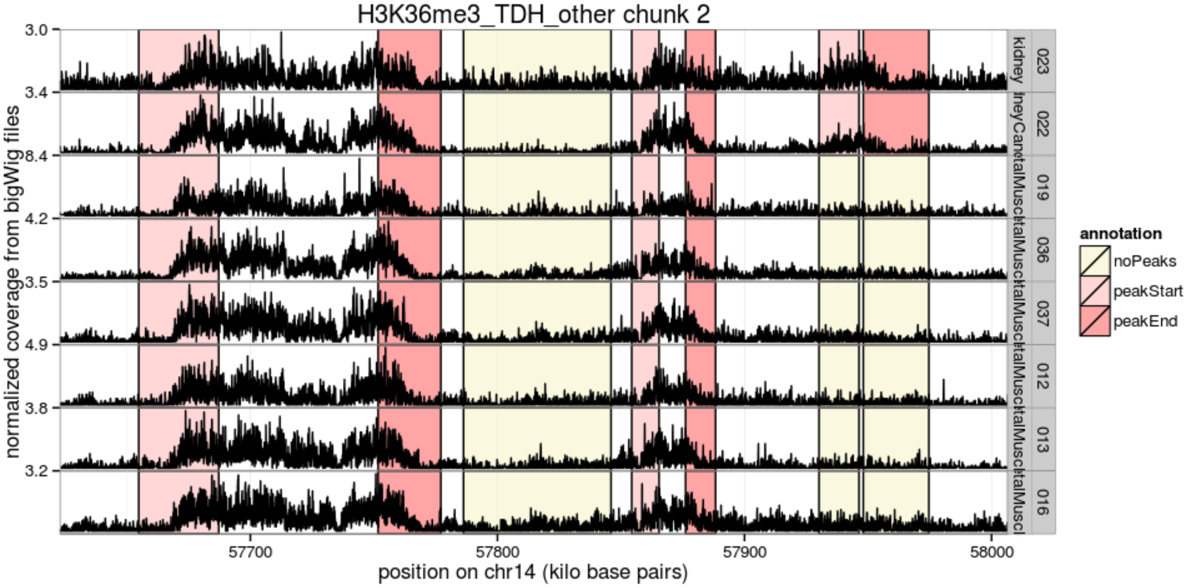

Labeling broad domains. Broad ChIP-seq marks such as H3K36me3 data are sometimes interpreted in terms of “domains” which may consist of one or more “peaks” perhaps separated by small regions of background. To label these data, there are several possibilities:

- If the data appear to be one broad peak covering the entire domain, then one large peakStart and one large peakEnd label can be used to indicate that one large peak should be called in this region (and not several smaller peaks).
- If the data appear to be several smaller peaks separated by small spaces, which visually make up a domain if concatenated, then such peaks would still be informative of the domain extension. In this

case, one small peakStart label and one small peakEnd label can be used to indicate that a consistent peak model should at least recover the start and end of the domain.

- If there is one (or very few) small peak(s) located somewhere in the domain, then the start and the end of the data may not be obvious. In this case, one can add peakStart and peakEnd labels to indicate that a small peak to be called, or perhaps not add any labels at all (if the interpretation of the peaks in the data is not clear).

Example of a labels file. Below is an example of an labels file that TDH created by manual visual inspection of the H3K4me3 immune cell samples (tcell, bcell, and monocyte). Genomic regions were copied from the UCSC genome browser web page, and pasted into a text file. It is divided into 4 genomic windows of nearby labels, each of which is separated by two returns/newlines. Each line represents a label for several cell types. The cell types that are not listed all get a noPeaks label in the indicated region. For example, the first line means that monocyte and tcell samples get a peakStart label at the indicated region, and the bcell samples get a noPeaks label in the same region.

```
chr11:118,092,641-118,095,026 peakStart monocyte tcell
chr11:118,095,334-118,096,640 peakEnd monocyte tcell
chr11:118,101,452-118,118,472 peaks
chr11:118,121,649-118,124,175 peaks monocyte tcell bcell

chr11:111,285,081-111,285,355 peakStart monocyte
chr11:111,285,387-111,285,628 peakEnd monocyte
chr11:111,299,681-111,337,593 peaks
chr11:111,635,157-111,636,484 peakStart monocyte tcell bcell
chr11:111,637,473-111,638,581 peakEnd monocyte tcell bcell

chr1:32,717,194-32,721,976 peaks tcell
chr1:32,750,608-32,756,699 peaks
chr1:32,757,261-32,758,801 peaks tcell bcell monocyte

chr2:26,567,544-26,568,406 peakStart bcell tcell monocyte
chr2:26,568,616-26,568,862 peakEnd bcell tcell monocyte
chr2:26,569,573-26,571,905 peakEnd bcell tcell monocyte
chr2:26,578,595-26,632,223 peaks
chr2:26,634,282-26,636,118 peaks monocyte
```

Supplementary Text 2

Details of peak calling algorithms. In the following paragraphs, we describe the details of the peak calling algorithms we tested.

We used Model-based Analysis of ChIP-Seq for the **macs** algorithm Zhang et al. [2008]. We downloaded MACS version 2.0.10 12162013 (commit ca806538118a85ec338674627f0ac53ea17877d9 on GitHub). We used the qvalue cutoff parameter `-q` for λ, with grid of 59 values from 0.8 to 10^{-15}. We used the `--broad` command line option for the **macs.broad** algorithm. With broad settings there are two separate qvalue cutoff parameters: (`-q` and `--broad-cutoff`). So we used the same grid of 59 qvalue cutoffs for `-q`, and then defined

$$\texttt{broad-cutoff} = \texttt{q} \times 1.122, \tag{1}$$

since `broad-cutoff` is required to be larger than `-q`. The default macs algorithms use a default qvalue cutoff of $\tilde{\lambda} = 0.05$.

We downloaded CCAT version 3.0 from `http://cmb.gis.a-star.edu.sg/ChIPSeq/paperCCAT.htm` Xu et al. [2010]. The `example/config_histone.txt` file was used for the **ccat.histone** algorithm, and the `example/config_TF.txt` file was used for the **ccat.tf** algorithm. We set minScore to 0 in both files, then analyzed the `significant.region` files after running CCAT. Column 7 in these files is the "fold-change score." We defined a grid of fold-change score thresholds $\lambda \in \{0.1, \dots, 400\}$, and we defined the CCAT model with parameter λ as all rows of the `significant.region` file which have a fold-change score greater than λ. The default CCAT algorithm uses a `minScore` of $\tilde{\lambda} = 5$.

The HMCan algorithm was proposed to correct for GC-content and copy number differences Ashoor et al. [2013]. We downloaded HMCan commit 9d0a330d0a873a32b9c4fa72c94d00968132b9ef from BitBucket. We used the default GC content normalization file provided by the authors. We used two different parameter files to test two different peak detectors:

name	mergeDistance
**hmcan**	200
**hmcan.broad**	1000

We then ran HMCan with finalThreshold=0, and defined λ as a threshold on column 5 in the regions.bed file. Default models use a finalThreshold of $\tilde{\lambda} = 10$.

We downloaded RSEG version 0.4.8 from `http://smithlabresearch.org/software/rseg/` Song and Smith [2011]. Upon recommendation of the authors, we saved computation time by running `rseg-diff` using options `-training-size 100000` and `-i 20`. We used the `-d` option to specify a dead regions file for hg19 based on our alignment pipeline. We defined the significance threshold λ as the sum of posterior scores (column 6 in the output .bed file). For the default RSEG algorithm, we used all the peaks in the output file, meaning a posterior score threshold of $\tilde{\lambda} = 0$.

We downloaded SICER version 1.1 from `http://home.gwu.edu/~wpeng/SICER_V1.1.tgz` Zang et al. [2009]. We defined the significance threshold λ as the FDR (column 8) in the islands-summary output file. For the default SICER algorithm, we used an FDR of $\tilde{\lambda} = 0.01$ as suggested in the README and example.

The HOMER set of tools was proposed for DNA motif detection Heinz et al. [2010]. We used the `findPeaks` program in HOMER version 4.1 with the `-style histone` option. We defined the significance threshold λ as the "p-value vs Control" column. We defined the default model as all peaks in the output file.

Supplementary Text 3

Definition of the label error. In this section we give the precise mathematical definition of the label error.

Data definition Let there be n labeled training samples, all of the same histone mark type. For simplicity, and without loss of generality, let us consider just one chromosome with b base pairs. Let $\mathbf{x}_1 \in \mathbb{Z}_+^b, \dots, \mathbf{x}_n \in \mathbb{Z}_+^b$ be the vectors of coverage across that chromosome. For example $b = 249, 250, 621$ is the number of base pairs on chr1, and $\mathbf{x}_i \in \mathbb{Z}_+^b$ is the H3K4me3 coverage profile on chr1 for one sample $i \in \{1, \dots, n\}$.

We also have exactly four sets of labeled regions $\underline{R}_i, \overline{R}_i, R_i^+, R_i^-$ for each sample $i \in \{1, \dots, n\}$:

Data	Type	Color	Symbols
Coverage			$\mathbf{x}_1, \dots \mathbf{x}_n$
Weak labels	peaks	purple	$R_1^+, \dots, R_n^+$
	noPeaks	light yellow	$R_1^-, \dots, R_n^-$
Strong labels	peakStart	pink	$\underline{R}_1, \dots, \underline{R}_n$
	peakEnd	red	$\overline{R}_1, \dots, \overline{R}_n$

For each sample $i \in \{1, \dots, n\}$, R_i^+ is a set of regions, and each region $\mathbf{r} \in R_i^+$ is an interval of base pairs, e.g. $\mathbf{r} = (100, 200)$.

A peak detection function or peak caller $c : \mathbb{Z}_+^b \to \{0, 1\}^b$ takes a coverage profile $\mathbf{x} \in \mathbb{Z}_+^b$ and returns a binary peak call prediction $\mathbf{y} = c(\mathbf{x}) \in \{0, 1\}^b$.

The goal is to learn how to call peaks $c(\mathbf{x}_i)$ which agree with the labeled regions $R_i^+, R_i^-, \underline{R}_i, \overline{R}_i$ for some test samples i. To quantify the error of the peak calls with respect to the labels, we define the following functions.

Weak label error The weak labels R_i^+, R_i^- count whether or not there are any peaks overlapping a region. They are called weak because each "peaks" label $\in R_i^+$ can only produce a false negative (but not a false positive), and each "noPeaks" label $\in R_i^-$ can only produce a false positive (but not a false negative). Fig. 3 shows how the "peaks" label counts false negatives and the "noPeaks" label counts false positives. Let

$$B(\mathbf{y}, \mathbf{r}) = \sum_{j \in \mathbf{r}} y_j \tag{2}$$

be the number of bases which have peaks overlapping region $\mathbf{r}$. Then for a sample i, the number of weak false positives is

$$\text{WFP}(\mathbf{y}, R_i^-) = \sum_{\mathbf{r} \in R_i^-} I\left[B(\mathbf{y}, \mathbf{r}) > 0\right], \tag{3}$$

where I is the indicator function. The weak false positive (WFP) function counts the number of "noPeaks" regions $\mathbf{r} \in R_i^-$ which have at least one overlapping peak.

The number of weak true positives is

$$\text{WTP}(\mathbf{y}, R_i^+) = \sum_{\mathbf{r} \in R_i^+} I\left[B(\mathbf{y}, \mathbf{r}) > 0\right]. \tag{4}$$

The weak true positive (WTP) function counts the number of "peaks" regions $\mathbf{r} \in R_i^+$ which have at least one overlapping peak.

Strong label error The strong labels $\underline{R}_i, \overline{R}_i$ count the number of peak starts and ends occuring in the given regions. They are called strong because each label can produce a false positive (more than one peak start/end predicted in the region) or a false negative (no peak start/end predicted). Fig. 3 shows how these "peakStart" and "peakEnd" labels count both false negatives and false positives.

First, let $y_0 = y_{b+1} = 0$ and define the set of first bases of all peaks as

$$\underline{\mathcal{I}}(\mathbf{y}) = \{j \in \{1, \dots, b\} : y_j = 1 \text{ and } y_{j-1} = 0\} \tag{5}$$

and the set of last bases of all peaks as

$$\overline{\mathcal{I}}(\mathbf{y}) = \{j \in \{1, \dots, b\} : y_j = 1 \text{ and } y_{j+1} = 0\}. \tag{6}$$

For a sample i, the number of strong false positives is

$$\text{SFP}(\mathbf{y}, \underline{R}_i, \overline{R}_i) = \sum_{\mathbf{r} \in \underline{R}_i} I\big[|\mathbf{r} \cap \underline{\mathcal{I}}(\mathbf{y})| > 1\big] + \sum_{\mathbf{r} \in \overline{R}_i} I\big[|\mathbf{r} \cap \overline{\mathcal{I}}(\mathbf{y})| > 1\big]. \tag{7}$$

The strong false positive (SFP) function counts the number of "peakStart" and "peakEnd" regions which contain more than one peak start/end.

The number of strong true positives is

$$\text{STP}(\mathbf{y}, \underline{R}_i, \overline{R}_i) = \sum_{\mathbf{r} \in \underline{R}_i} I\big[|\mathbf{r} \cap \underline{\mathcal{I}}(\mathbf{y})| > 0\big] + \sum_{\mathbf{r} \in \overline{R}_i} I\big[|\mathbf{r} \cap \overline{\mathcal{I}}(\mathbf{y})| > 0\big]. \tag{8}$$

The strong true positive (STP) function counts the number of "peakStart" and "peakEnd" regions which contain at least one peak start/end.

Total label error For a sample i, the total number of false positives is

$$\text{FP}(\mathbf{y}, \underline{R}_i, \overline{R}_i, R_i^-) = \text{WFP}(\mathbf{y}, R_i^-) + \text{SFP}(\mathbf{y}, \underline{R}_i, \overline{R}_i), \tag{9}$$

the total number of true positives is

$$\text{TP}(\mathbf{y}, \underline{R}_i, \overline{R}_i, R_i^+) = \text{WTP}(\mathbf{y}, R_i^+) + \text{STP}(\mathbf{y}, \underline{R}_i, \overline{R}_i), \tag{10}$$

the total number of false negatives is

$$\text{FN}(\mathbf{y}, \underline{R}_i, \overline{R}_i, R_i^+) = |\underline{R}_i| + |\overline{R}_i| + |R_i^+| - \text{TP}(\mathbf{y}, \underline{R}_i, \overline{R}_i, R_i^+). \tag{11}$$

The label error E quantifies the number of incorrect labels:

$$E(\mathbf{y}, \underline{R}_i, \overline{R}_i, R_i^+, R_i^-) = \text{FP}(\mathbf{y}, \underline{R}_i, \overline{R}_i, R_i^-) + \text{FN}(\mathbf{y}, \underline{R}_i, \overline{R}_i, R_i^+). \tag{12}$$

The label error E can be easily computed using the C code in the R package PeakError on GitHub: `https://github.com/tdhock/PeakError`.

ROC analysis A peak caller with a scalar parameter $\lambda \in \mathbb{R}$ that controls the number of peaks detected can be characterized as a function $c_\lambda : \mathbb{Z}_+^b \rightarrow \{0,1\}^b$. Receiver Operating Characteristic (ROC) curves can be used to show how the true positive and false positive rates vary as a function of λ. Define the false positive rate as

$$\text{FPR}(\lambda) = \frac{\sum_{i=1}^n \text{FP}\left[c_\lambda(\mathbf{x}_i), \underline{R}_i, \overline{R}_i, R_i^-\right]}{|\underline{R}_i| + |\overline{R}_i| + |R_i^-|} \tag{13}$$

and the true positive rate as

$$\text{TPR}(\lambda) = \frac{\sum_{i=1}^n \text{TP}\left[c_\lambda(\mathbf{x}_i), \underline{R}_i, \overline{R}_i, R_i^+\right]}{|\underline{R}_i| + |\overline{R}_i| + |R_i^+|}. \tag{14}$$

ROC curves are traced by plotting $\text{TPR}(\lambda)$ versus $\text{FPR}(\lambda)$ for all possible values of the peak detection parameter λ.

Supplementary Figure 1

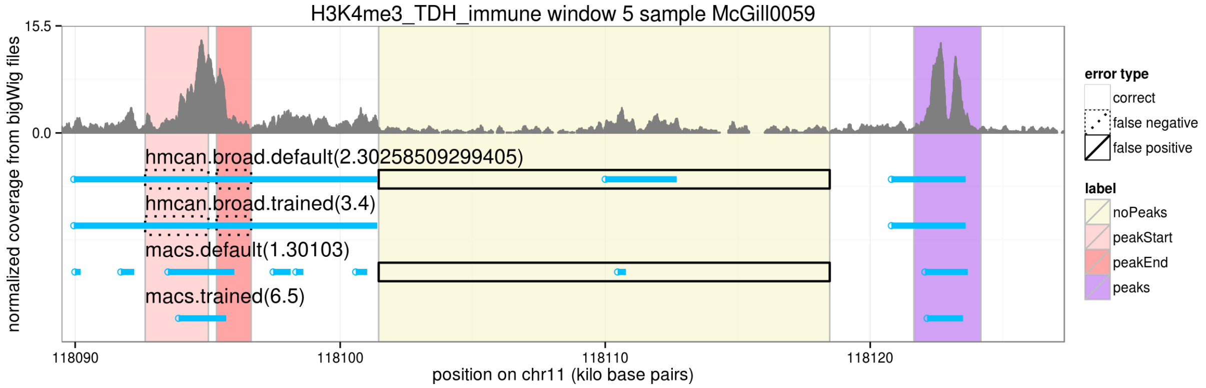

Default and learned peak detectors in H3K4me3 data. It is clear that default parameter values yield false negatives and false positives. Parameter training for macs yields a perfect peak detection model.

Supplementary Figure 2

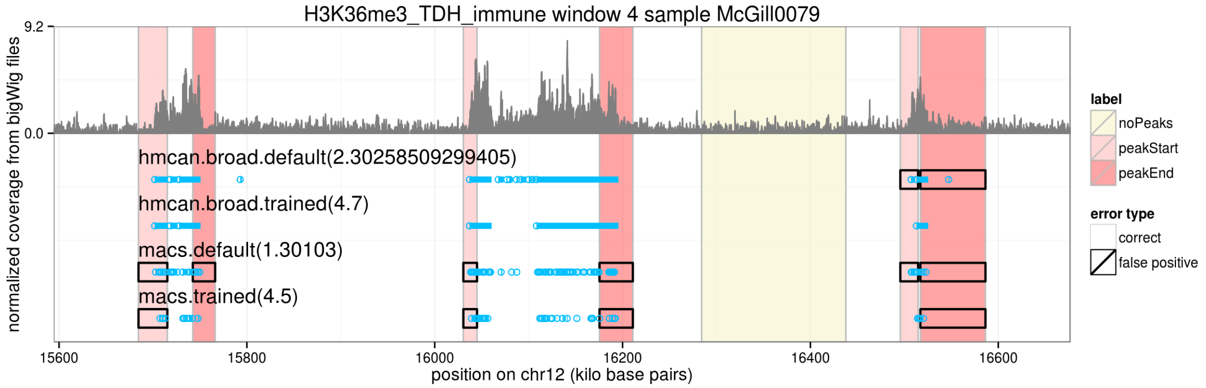

Default and learned peak detectors in H3K36me3 data. It is clear that default parameter values yield false positives. Parameter training for hmcan.broad yields a perfect peak detection model.

Supplementary Figure 3

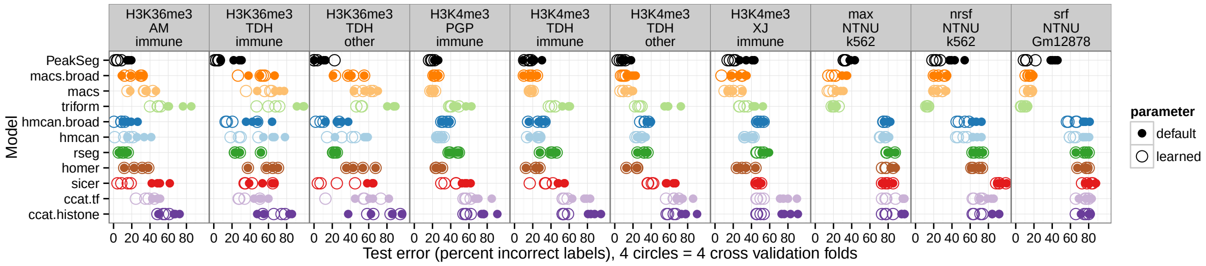

Test error for all algorithms and all data sets. It is clear that each unsupervised algorithm yields accurate peak detection for specific experiments. For example, triform is accurate in transcription factor data sets (max, nrsf, srf) but not in histone marks. In contrast, the supervised PeakSeg method yields state-of-the-art accuracy in all data sets (except max).

Supplementary Figure 4

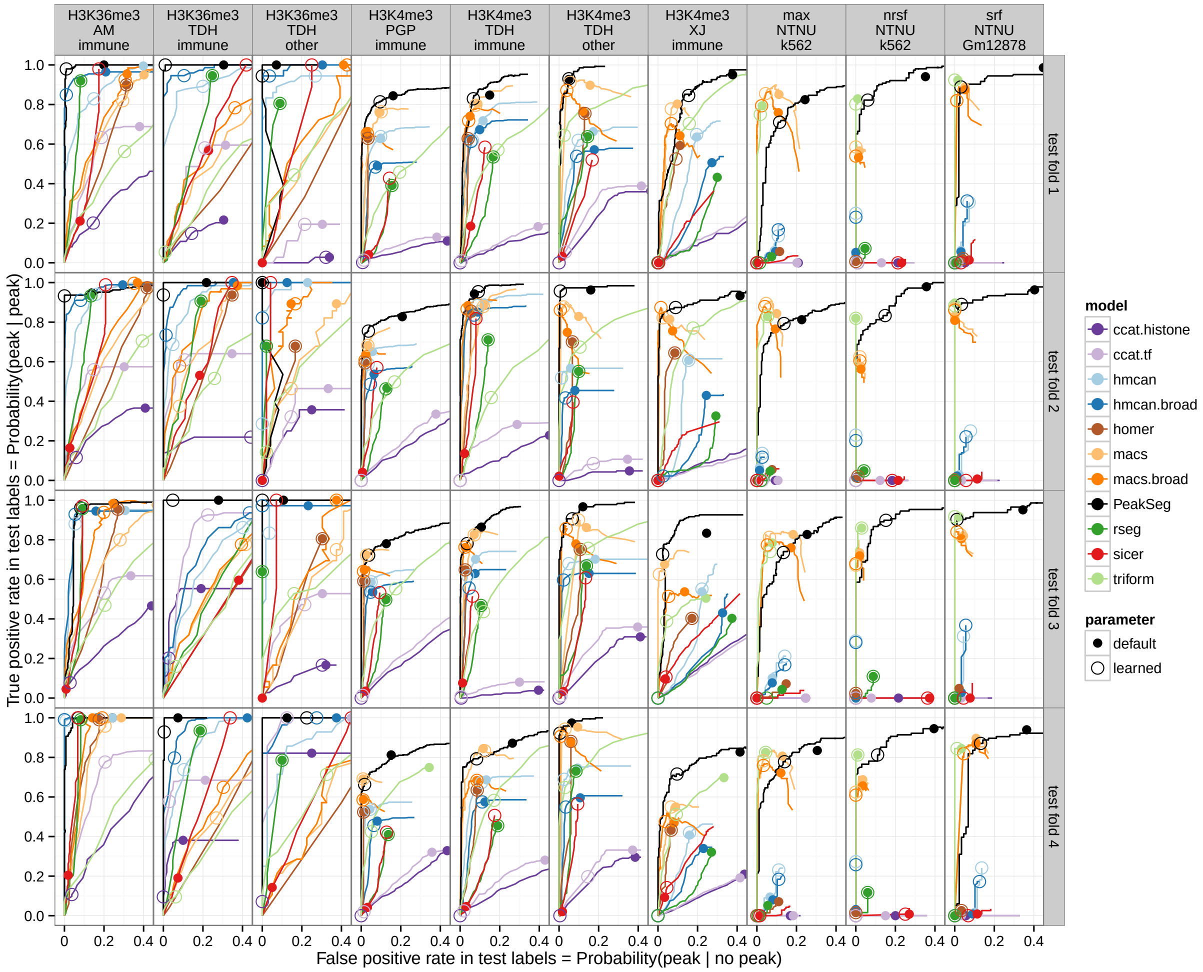

ROC curves for 4-fold cross-validation test error, for all algorithms and all data sets. Unlike usual ROC curves, these are not monotonic, since some of the parameters (e.g. the macs qvalue parameter) affect peak size as well as the number of peaks. It is clear that default parameters tend to have higher false positive rates than learned parameters. It is also clear that some algorithms are better than others for all possible thresholds.

Supplementary Figure 5

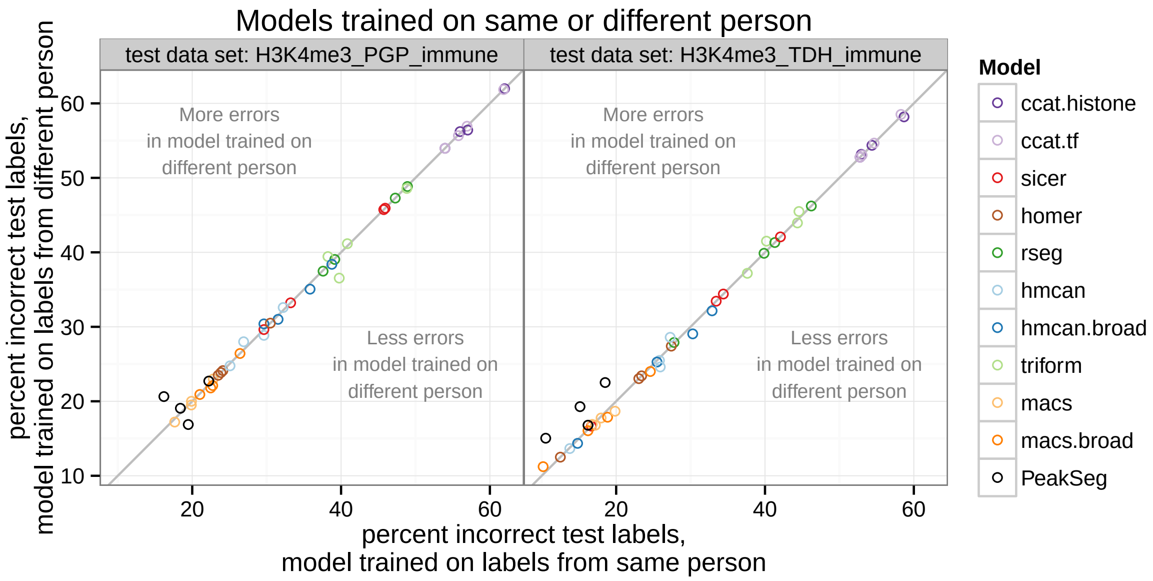

Train on one person, test on another. H3K4me3 immune cell data sets were labeled by TDH and PGP. We trained models using labels from one or the other person, and then tested those models on labels from the same or a different person. It is clear for all models that it does not make much difference in terms of test error when training using labels from one or another person.

Supplementary Figure 6

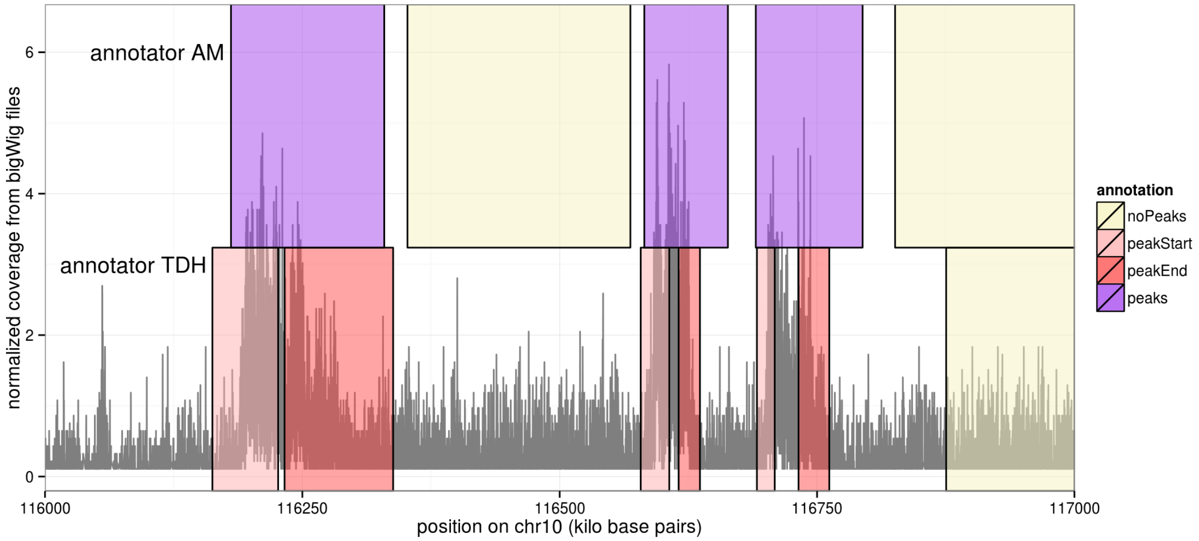

Windows labeled by 2 different people. Labels for 2 different annotators (top=AM, bottom=TDH) on the same genomic regions and sample sets (H3K36me3 immune cell types). It is clear that the people focus on different levels of detail, but have in general the same visual definition of a peak.

Supplementary Figure 7

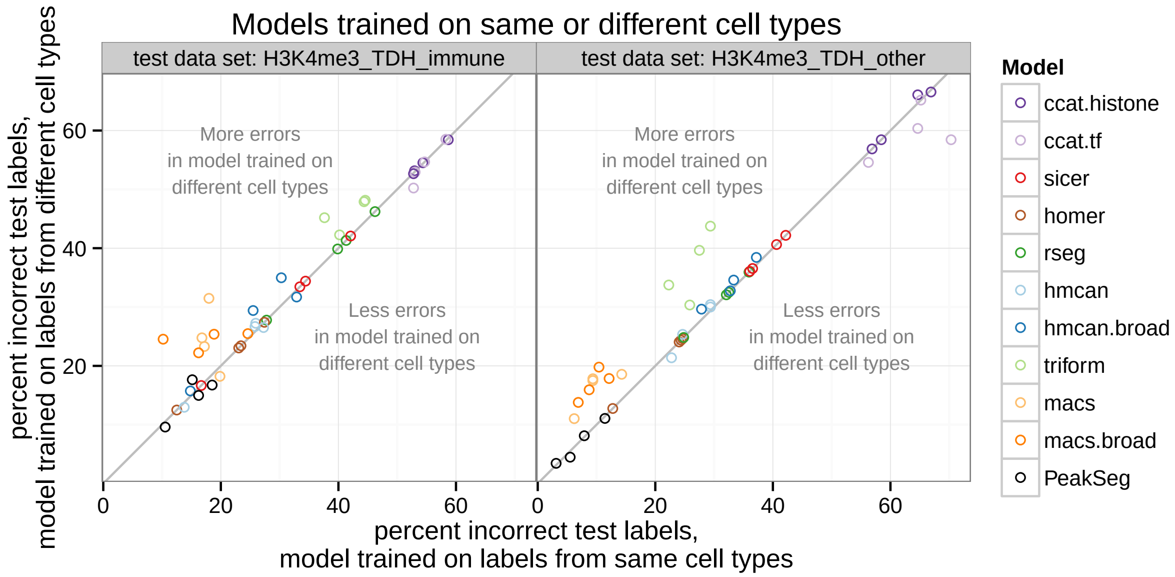

Train on some cell types, test on others. TDH labeled H3K4me3 data sets of immune and other cell types. We trained models on one or the other cell types, and then tested those models on the same or a different set of cell types. It is clear that for some models such as macs and macs.broad, a model trained on the different cell types yields higher test error than a model trained on the same cell types. It is also clear that the train data do not make much difference for other models.

Supplementary Figure 8

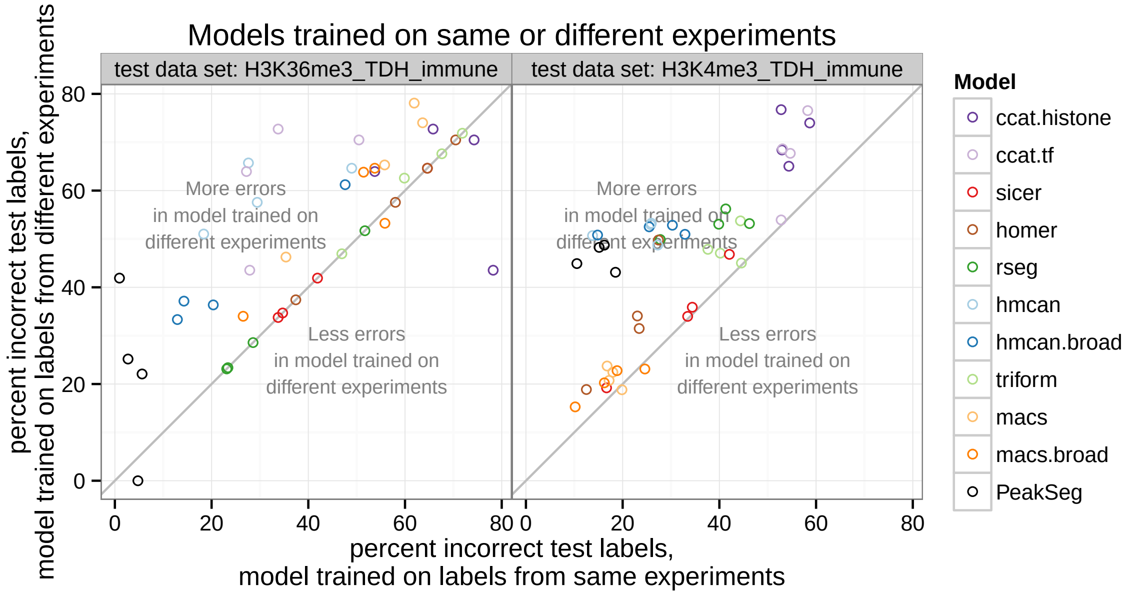

Train on one histone mark, test on another. TDH labeled H3K4me3 and H3K36me3 profiles for the same set of immune cell samples. We trained models on one or the other histone mark, and then tested those models on the same or a different histone mark. It is clear that a model trained on a different histone mark yields higher test error than a model trained on the same histone mark.

Supplementary Table 1

experiment	H3K36me3	H3K36me3	H3K36me3	H3K4me3	H3K4me3	H3K4me3	H3K4me3	max	nrsf	srf
annotator	AM	TDH	TDH	PGP	TDH	TDH	XJ	NTNU	NTNU	NTNU
cell.types	immune	immune	other	immune	immune	other	immune	k562	k562	Gm12878
windows	23	4	4	30	27	29	12	207	191	171
noPeaks	752	230	72	1653	1656	536	702	248	264	184
peaks	403			638	287	218	243			
peakStart	294	200	68	813	796	305	216	452	276	270
peakEnd	294	200	60	730	933	311	216	452	276	270
tcell	15	15		19	19		19			
monocyte	5	5		6	6		6			
bcell	1	1		2	2		2			
kidney			1			1				
kidneyCancer			1			1				
skeletalMuscleCtrl			3			3				
skeletalMuscleMD			3			4				
leukemiaCD19CD10BCells						1				
k562								2	2	
Gm12878										2

Counts of labeled genomic windows, label types, and samples of each cell type in each of the 10 data sets that we analyzed.

Supplementary Table 2

Peaks:	0	1	2	3	4	5	6	7	8	9	mean	samples	windows
H3K36me3_AM_immune	24	232	159	22	9	9	6	10	1	11	1.88	21	23
H3K36me3_TDH_immune	1	18	32	10	2	19	2	0	0	0	2.70	21	4
H3K36me3_TDH_other	0	0	9	11	1	2	4	2	0	3	4.06	8	4
H3K4me3_PGP_immune	88	261	200	124	73	31	24	4	3	2	2.09	27	30
H3K4me3_TDH_immune	0	184	164	126	108	48	36	19	11	33	3.15	27	27
H3K4me3_TDH_other	0	35	73	65	40	32	16	6	5	18	3.57	10	29
H3K4me3_XJ_immune	1	137	133	30	12	6	4	1	0	0	1.86	27	12
max_NTNU_k562	69	121	103	68	25	15	6	3	2	2	1.93	2	207
nrsf_NTNU_k562	105	231	30	11	4	1	0	0	0	0	0.90	2	191
srf_NTNU_Gm12878	86	205	35	14	2	0	0	0	0	0	0.95	2	171

Counts of peaks per sample-window predicted by the PeakSeg model in the labeled genomic windows of the ten data sets that we analyzed. For example, the first row of the table shows that the H3K36me3_AM_immune data set contains 21 samples, 23 windows, 24 sample-windows with no predicted peaks, and a mean of 1.88 predicted peaks per sample-window.

Acknowledgements

This work was supported by computing resources provided by Calcul Quebec and Compute Canada, National Sciences and Engineering Council of Canada RGPGR 448167-2013, and by Canadian Institutes of Health Research grants EP1-120608 and EP1-120609, awarded to GB.

References

H. Ashoor, A. Hérault, A. Kamoun, F. Radvanyi, V. B. Bajic, E. Barillot, and V. Boeva. HMCan: a method for detecting chromatin modifications in cancer samples using ChIP-seq data. *Bioinformatics*, 29(23):2979–2986, 2013.

S. Heinz, C. Benner, N. Spann, E. Bertolino, Y. C. Lin, P. Laslo, J. X. Cheng, C. Murre, H. Singh, and C. K. Glass. Simple Combinations of Lineage-Determining Transcription Factors Primecis-Regulatory Elements Required for Macrophage and B Cell Identities. *Molecular cell*, 38(4):576–589, 2010.

Q. Song and A. D. Smith. Identifying dispersed epigenomic domains from chip-seq data. *Bioinformatics*, 27(6):870–871, 2011.

H. Xu, L. Handoko, X. Wei, C. Ye, J. Sheng, C.-L. Wei, F. Lin, and W.-K. Sung. A signal-noise model for significance analysis of ChIP-seq with negative control. *Bioinformatics*, 26(9):1199–1204, 2010.

C. Zang, D. E. Schones, C. Zeng, K. Cui, K. Zhao, and W. Peng. A clustering approach for identification of enriched domains from histone modification ChIP-Seq data. *Bioinformatics*, 25(15):1952–1958, 2009.

Y. Zhang, T. Liu, C. A. Meyer, J. Eeckhoute, D. S. Johnson, B. E. Bernstein, C. Nusbaum, R. M. Myers, M. Brown, W. Li, et al. Model-based analysis of ChIP-Seq (MACS). *Genome Biol*, 9(9):R137, 2008.
